# Outcome expectations and repeated blood donation behavior: a moderated mediation model in a prospective observational study

**DOI:** 10.1093/abm/kaaf036

**Published:** 2025-05-21

**Authors:** Huahua Hu, Wei Hu, Joseph Tak Fai Lau, Qiuyue Hu, Yan Yan, Phoenix Kit Han Mo

**Affiliations:** Blood Center of Zhejiang Province, Hangzhou, 310052, China; Blood Center of Zhejiang Province, Hangzhou, 310052, China; Public Mental Health Center, School of Mental Health, Wenzhou Medical University, Wenzhou, 325035, China; Zhejiang Provincial Clinical Research Center for Mental Disorders, The Affiliated Wenzhou Kangning Hospital, Wenzhou Medical University, Wenzhou, 325088, China; Blood Center of Zhejiang Province, Hangzhou, 310052, China; Blood Center of Zhejiang Province, Hangzhou, 310052, China; School of Public Health and Primary Care, The Chinese University of Hong Kong, 999077, Hong Kong; CUHK Centre for Public Health and Primary Care (Shenzhen), Shenzhen Research Institute of the Chinese University of Hong Kong, Shenzhen, 518172, China

**Keywords:** outcome expectations, anticipated physical benefits, altruism, re-donation intention, repeated blood donation behavior, moderated mediation

## Abstract

**Background:**

Social cognitive theory proposes outcome expectations as an important factor in behavior change and maintenance. However, it is unknown whether and how outcome expectations interact with prosocial traits in predicting repeated blood donation (RBD) behavior.

**Purpose:**

The current study aimed to test the prospective association between outcome expectations and RBD behavior and the roles of altruism and re-donation intention in this association.

**Methods:**

A total of 850 blood donors recruited from blood donation sites in Hangzhou, China, completed an online baseline survey. Their RBD behavior was captured by checking their blood donation records in the database of Blood Center of Zhejiang Province 6 months later.

**Results:**

Anticipated physical benefits positively predicted RBD behavior, while anticipated physical harms negatively predicted it. Moderated mediation analyses showed that overall outcome expectations and anticipated physical benefits predicted more RBD behavior through increased re-donation intention, and this effect was moderated by altruism. Specifically, the positive effect of overall outcome expectations and anticipated physical benefits on re-donation intention was stronger among donors with lower levels of altruism. Overall outcome expectations and anticipated physical benefits were found to have a significant impact on RBD behavior only in donors with lower levels of altruism.

**Conclusions:**

The findings suggest that interventions aimed at promoting RBD behavior should seek to enhance re-donation intention, overall outcome expectations, and altruism. In particular, it is important to increase the overall outcome expectations—especially anticipated physical benefits—of donors with lower levels of altruism.

## Introduction

Retaining blood donors and encouraging repeated blood donation (RBD) are essential for ensuring a stable and sufficient supply of blood for transfusion.^1^ RBD refers to the process in which donors return to donate blood twice or more.^2^ Outcome expectations, defined as the perceived positive or negative consequences of performing a specific action,^3^ have been shown to influence RBD intention—but not RBD behavior—among Australian blood donors.^4^ It should, however, be noted that this study is a cross-sectional survey,^4^ which may have influenced the reliability of the results. Specifically, the cultural context may have played a significant role in shaping the observed relationship between outcome expectations and RBD intention or behavior. In China, characterized by a high level of collectivism,^5^ personal beliefs (eg, outcome expectations) may have weaker influences on behavior than in individualistic countries such as Australia.^6^ It is important to examine the prospective association between outcome expectations and RBD behavior in China to understand the roles of these factors in different cultural contexts. Furthermore, research on the mechanisms underlying the relationship between outcome expectations and prosocial personality factors with RBD is scarce. In this study, we hypothesize that there exists a moderated mediation effect in which altruism moderates the relationship between outcome expectations, RBD intention, and RBD behavior. We test this effect using a prospective observational design. The findings may help address a novel and important research gap regarding how altruism interacts with outcome expectations to influence the social-cognitive functioning of RBD. Such results will benefit the development of RBD interventions and help blood collection agencies and policymakers identify the specific group that is most likely to benefit from relevant intervention programs.

### The relationship between outcome expectations and RBD behavior

According to social cognitive theory (SCT), outcome expectations play an important role in the motivational process underlying behavior outcomes.^7^ Other prominent theories, such as the health belief model, theory of planned behavior, and health action process approach also propose that individuals modify their behavior based on their assessment of the health outcomes, including potential benefits and harms, of the particular behavior.^8-10^ It is well understood that individuals learn from and are motivated by outcome expectations of certain actions. They tend to engage in behaviors that they believe will bring positive outcomes while avoiding those that may result in negative or undesirable consequences.^8,9,11^ In the context of blood donation, positive outcome expectations (eg, perceived benefits of donating) are likely to increase donation intention and behavior, while negative outcome expectations (eg, anticipated physical harm or adverse reactions) may reduce them.^4,12,13^ Although previous studies have linked outcome expectations to re-donation intention,^4,12,13^ the direct relationship between outcome expectations and RBD behavior is understudied. A previous study has indicated that anticipated physical harms (eg, possible infection, adverse reactions) are significant barriers to repeated donation.^14^ Therefore, it is possible that outcome expectations would predict RBD behavior, with positive expectations associated with higher engagement and negative expectations with lower engagement in RBD.

### The mediation role of re-donation intention

Outcome expectations may influence RBD behavior through re-donation intention. Theoretical and empirical studies have indicated that intention is a crucial determinant of behavior and acts as a full mediator of the association between other factors and behavior. For instance, the theory of planned behavior and the closely related reasoned action approach suggest that intention is the central construct and a direct determinant of behavior,^10,15^ while attitude, subjective norm, and perceived control are immediate determinants of behavior intention.^10^ Empirical studies have reported that re-donation intention mediated the relationship between attitude and RBD behavior^16,17^; however, no studies have explicitly tested the mediation role of re-donation intention in the relationship between outcome expectations and RBD behavior. Outcome expectations and attitudes are related constructs that reflect the individual's overall evaluation of a behavior.^7,10^ It is reasonable to hypothesize that outcome expectations would predict RBD behavior through re-donation intention.

### The moderation role of altruism

Altruism refers to the desire/tendency to help/benefit others without any prospect of compensation.^18,19^ Previous literature has suggested that personality factors (eg, altruism, conscientiousness) are moderators that amplify or mitigate the impact of cognitive factors (eg, attitude, self-efficacy) on behavior intentions.^20,21^ Specifically, altruistic individuals may prioritize the well-being of others over personal benefits, resulting in a potentially weaker association between outcome expectations and re-donation intention in such individuals.^22^ While no studies have directly examined the moderating role of altruism in this context, indirect evidence indicates that altruism moderates the positive association between attitude/subjective norm and ecopreneurship intention (ie, intention to start a career involving environmentally friendly activities).^23^ Therefore, it is possible that altruism would moderate the association between outcome expectations and re-donation intention.

**Figure 1.**
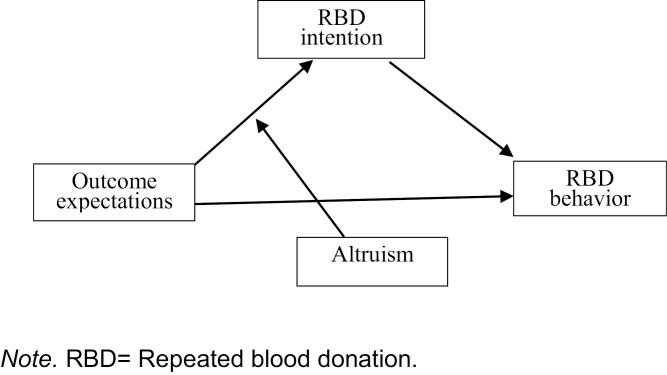
Conceptual model for the moderation effect of altruism in the influence of outcome expectations on repeated blood donation behavior through re-donation intention. RBD = repeated blood donation.

We propose a moderated mediation model (see Figure 1) to examine the role of altruism—through re-donation intention—in the relationships between outcome expectations and RBD behavior. The hypotheses are as follows:

H1. Overall outcome expectations predict RBD behavior.

H1a. Positive outcome expectations predict higher engagement in RBD behavior.

H1b. Negative outcome expectations predict lower engagement in RBD behavior.

H2. Re-donation intention mediates the effect of outcome expectations on RBD behavior.

H3. Altruism moderates the positive association between outcome expectations and re-donation intention. Specifically, the association between outcome expectations and re-donation intention is stronger for donors with lower (relative to higher) altruism.

## Method

### Inclusion/exclusion criteria

The inclusion criteria of the participants were: (1) voluntary, non-remunerated whole-blood donors who have donated once or more since 2018; (2) eligible for blood donation (healthy; aged 18-55 years; weight ≥50 kg for males and ≥45 kg for females; normal blood pressure; no AIDS, hepatitis B, or other infectious diseases; no other diseases that are not suitable for blood donation). The exclusion criteria were: (1) group donors whose past donations had all been organized by institutions; (2) regular blood donors who had 3 or more donations in total, and donated once or more in the previous year; (3) donors who had difficulties understanding Chinese.

### Procedure

A prospective observational study was conducted, comprising a 15-minute online survey at baseline and tracking of RBD behavior at a 6-month follow-up. Eligible donors were identified in a database of 682,480 donors by a staff of Blood Center of Zhejiang Province in Apr 2020.

Participants were stratified into 6 tiers based on the time of their past donation behavior. We expected to choose 3200 donors from each tier in order to achieve the targeted sample size. Because only 2967 donors could be located for one tier of donors in the database, a total of 18967 donors (i.e., 3200 donors from 5 tiers and 1976 donors from 1 tier) were selected randomly from the database of Blood Center of Zhejiang Province (see Table 1 in supplementary materials). Selected donors were contacted by the short message service (SMS) platform of Blood Center of Zhejiang Province, informed about the study's background and purpose, and invited to complete the online survey via a provided link. Those who accessed the link were presented with detailed study information and asked to provide informed consent before proceeding. A total of 1493 participants accessed the online survey between 29 March and 9 May 2021, and 1025 met the inclusion criteria and completed the questionnaire. Two data screening criteria—instructed and self-report items^24^—were used for quality checks. The instructed item asked participants to choose a particular option^24^; the self-report item asked participants to rate the extent to which they answered all questions carefully.^25^ A total of 175 responses were detected as invalid based on these 2 criteria and further excluded. Consequently, 850 valid questionnaires were included for analysis. At the 6-month follow-up (November 2021), the re-donation behavior of these donors was checked using the database of Blood Center of Zhejiang Province. Ethics approval was obtained from the Ethics Committee of The Chinese University of Hong Kong.

### Measurements

#### Outcome expectations

Outcome expectations were assessed using an outcome expectation scale previously developed and validated by the team.^13^ This scale consists of 5 dimensions: anticipated physical benefits (3 items), social or tangible gains (6 items), self-worth expectancy (3 items), physical harms (3 items), and unfavorable social expectancies (3 items). The former 3 dimensions are positive outcome expectations, while the latter 2 are negative outcome expectations. Donors were invited to rate their agreement with the statements about the potential results or consequences of donating blood. Sample items include, “Donating blood would be good for health,” “I would feel a sense of accomplishment after donating blood,” and “I would experience adverse reactions after donating blood.” Responses ranged from 1 = Strongly disagree to 5 = Strongly agree, with higher scores reflecting greater levels of the respective outcome expectations. The overall outcome expectations score was computed by subtracting the total score of the negative dimensions from that of the positive dimensions. The Cronbach’s alpha of this scale was 0.86.

#### Altruism

Altruism was assessed using the altruism subscale of the prosocial tendency scale.^26^ This scale has been validated and used in Chinese samples and among adults.^27-29^ It consists of 4 items measuring helping tendency when a direct, explicit reward is not perceived to be possible for oneself. Sample items include “I often assist even if I have no expectation of obtaining anything in return.” and “I donate goods/money but not for the purpose of benefiting from it.” Responses ranged from 1 = Strongly disagree to 5 = Strongly agree, with higher scores indicating higher levels of altruism. The Cronbach’s *α* for this scale was 0.85.

#### Re-donation intention

Re-donation intention was assessed by adapting the items from the 2-item donation intention scale for money donation.^30^ In the current study, RBD was used to replace monetary donation; for example, through the item “I intend to donate blood within the next 12 months.” Responses ranged from 1 = Not at all likely to 5 = Very much likely, with higher scores indicating higher levels of re-donation intention. The Spearman’s rank correlation was 0.91 in the present study.

#### Re-donation behavior

RBD behavior was measured at 6-month follow-up by checking the donation record (successful donation) through the database of Blood Center of Zhejiang Province. Participants with a donation record during the 6-month follow-up were coded 1, while those without were coded 0.

### Statistical analyses

To examine the correlations among the background variables and study variables, Spearman and Pearson correlation analyses were conducted using IBM SPSS version 21.0. The background variables (eg, BMI, age, marital status, occupation, education level, frequency of past donation) that were significantly associated with re-donation intention and RBD behavior were treated as covariates in further analyses. By using Mplus 8.0, regression analysis was conducted to test H1. Mediation analyses were performed to test H2 using structural equation modeling (SEM) and bootstrapping analysis (*n* = 2000). Statistical significance was determined using 95% confidence intervals that excluded zero. Considering how the score of overall outcome expectations was calculated, H3 was tested in a moderated mediation model using overall outcome expectations as a predictor (the primary model and analysis) and in 5 exploratory models (analyses) using each subscale as a predictor. Mplus 8.0 was used for the SEM models. The study variables entered in the analyses were treated as manifest variables. To deal with the binary outcome, a diagonal weighted least squares robust estimation technique of weighted least squares—mean and variance adjusted (WLSMV) estimator was used, which generated probit estimates.^31^ This estimator outperforms other estimation methods (eg, maximum likelihood) when dealing with categorical and non-normal data.^32-34^ The study variables (except for the outcome variable RBD behavior) were converted to *Z*-scores for analyses. The significance *P*-value was 0.05.

## Results

### Sample characteristics

Of the 850 participants, more than half were 18-25 years old, and only 10% were aged 36 years and older. Approximately 64% of the participants had a BMI of 18.5-23.9. More than half of the participants were male. The majority of the participants were single. More than one-third of them had a monthly income of 2000 or less RMB, while 42% earned 4001 or more RMB every month. About half of the participants obtained a college level of education, and 47% were local residents with household registration in Hangzhou. Half of the donors were students or unemployed. About one-third of the participants were first-time donors at baseline. More than half of them had donated twice (see Table 1).

**Table 1:** Demographic and donation characteristics of the participants (*n* = 850).

	*N*	% (row)
Age		
18-25	583	69%
26-35	179	21%
36 and order	88	10%
BMI		
Less than 18.5	59	7%
18.5-23.9	542	64%
24-27.9	202	24%
28 or more	47	5%
Gender		
Female	390	46%
Male	460	54%
Marriage status		
Single	654	77%
Married/divorced/widowed	196	23%
Monthly income (Chinese Yuan)		
2000 or less	316	37%
2001-3000	88	10%
3001-4000	86	10%
4001 or more	360	43%
Education level		
High school or below	96	11%
Tertiary	250	29%
College	448	53%
Master or above	56	7%
Residence		
Non-native	450	53%
Native	400	47%
Occupation		
Student or unemployment	423	50%
Employees in company	226	27%
Individual practitioner or freelancer or farmer	89	10%
Military/police or civil servant or teacher	44	5%
Medical staff or lawyer	40	5%
Other	28	3%
Number of past donations		
1	274	32%
2	454	53%
3-4	122	14%

*Note.* Native refers to those who have household registration in Hangzhou.

Individual practitioner is a kind of occupation in which the employer has employed only 8 or fewer people.

### Correlations among the study variables

As shown in Table 2, overall outcome expectations, anticipated physical benefits, anticipated social or tangible gains, and self-worth expectancy were positively associated with altruism and re-donation intention. Anticipated physical harms and unfavorable social expectancies were negatively associated with altruism and re-donation intention. Overall outcome expectations, anticipated physical benefits, and self-worth expectancy were positively associated, while anticipated physical harms were negatively associated with re-donation behavior at 6-month follow-up. Altruism was significantly and positively associated with re-donation intention.

**Table 2:** Correlations among the study variables

	1	2	3	4	5	6	7	8
1. Overall outcome expectations	1							
2. Anticipated physical benefits	0.63^***^	1						
3. Anticipated social or tangible gains	0.61^***^	0.52^***^	1					
4. Self-worth expectancy	0.59^***^	0.39^***^	0.59^***^	1				
5. Anticipated physical harms	−0.69^***^	−0.27^***^	−0.12^***^	−0.22^***^	1			
6. Unfavorable social expectancies	−0.73^***^	−0.24^***^	−0.13^***^	−0.19^***^	0.41^***^	1		
7. Altruism	0.30^***^	0.22^***^	0.17^***^	0.29^***^	−0.20^***^	−0.18^***^	1	
8. Re-donation intention	0.39^***^	0.35^***^	0.25^**^	0.31^***^	−0.24^***^	−0.22^***^	0.28^***^	1
9. Follow up RBD behavior	0.08^*^	0.09^**^	0.06	0.09^**^	−0.10^**^	0.01	0.06	0.29^***^

*Note.* Spearman/Pearson’s correlation analyses; follow-up repeated blood donation behavior was dummy coded in the analyses. Abbreviation: RBD = Repeated blood donation.

^*^ *P* < .05; **P < .05; ***P < .001.

### Mediation role of re-donation intention

As displayed in Table 3, regression analysis indicated that overall outcome expectations did not significantly predict RBD behavior when significant background variables were included in the analysis, which was inconsistent with H1. Considering that the mediation effect may still exist when the direct effect is non-significant,^35^ we constructed an SEM model for the mediation effect of re-donation intention in the relationship between overall outcome expectations and RBD behavior. The model displayed a satisfactory model fit: χ^2^/df = 2.01, CFI = 0.98, TLI = 0.93, RMSEA = 0.03 (0.00, 0.06), SRMR = 0.02. As hypothesized (H2), higher overall outcome expectations were significantly associated with increased re-donation intention (*B* = 0.36), which in turn significantly predicted more RBD behavior (*B = *0.20). The indirect effect of overall outcome expectations on behavior through intention was significant (*B = *0.07, 95%CI, 0.05-0.09). The direct and total effect of overall outcome expectations on RBD behavior was non-significant.

**Table 3:** Mediation effect of outcome expectations on repeated blood donation behavior via re-donation intention.

	Equation 1 (RBD behavior)		Equation 2 (RBD intention)		Equation 3 (RBD behavior)	
	*β*	95% CI	*β*	95% CI	*β*	95% CI
RBD intention					0.20	0.15, 0.26
Outcome expectations	0.08	−0.01,0.17	0.36	0.30, 0.42	−0.02	−0.09, 0.05
BMI	0.11	0.02,0.20	0.09	0.02, 0.14	0.06	−0.01, 0.12
Married	0.11	0.02,0.20	0.03	−0.05, 0.10	0.06	−0.01, 0.14
NPD	0.35	0.26,0.43	0.27	0.22, 0.32	0.21	0.14, 0.27
IPPF	0.04	−0.06,0.12			0.04	−0.03, 0.12
Age			0.01	−0.04, 0.05		
2000 or less monthly income			0.01	−0.08, 0.10		
4001 or more monthly income			−0.02	−0.11, 0.07		
High school or below education			0.03	−0.03, 0.08		
Student or unemployment			−0.09	−0.19, 0.02		
Residence			0.07	0.01, 0.14		
*R* ^2^	0.161		0.246		0.123	
*F*	22.83		17.31		15.17	

Abbreviations: NPD = number of past donations; IPFF = Individual practitioner or freelancer or farmer. RBD = repeated blood donation.

Exploratory analyses evaluating the predictive role of the 5 outcome expectation subscales showed that the anticipated physical benefits predicted more (*B* = 0.10) while the anticipated physical harms predicted less RBD behavior (*B* = − 0.10). In addition, anticipated social or tangible gains and self-worth expectancies indirectly predicted more RBD behavior through increased re-donation intention, while unfavorable social expectancies indirectly predicted less RBD behavior through decreased re-donation intention (see Table 4).

**Table 4:** Mediation effect of the 5 dimensions of outcome expectations on repeated blood donation behavior via re-donation intention.

	Equation 1 (RBD behavior)			Equation 2 (RBD intention)			Equation 3 (RBD behavior)		
	*β*	*P*	95% CI	*β*	*P*	95% CI	*β*	*P*	95% CI
PPOE	0.10	.03	0.01, 0.20	0.33	.00	0.27, 0.38	0.00	.92	−0.06, 0.08
SPOE	0.06	.19	−0.03, 0.14	0.25	.00	0.19, 0.31	−0.01	.90	−0.08, 0.06
SEPOE	0.09	.06	−0.00, 0.18	0.29	.00	0.23, 0.36	0.00	.99	−0.07, 0.06
PNOE	−0.10	.03	−0.19, − 0.02	−0.21	.00	−0.27, − 0.15	−0.04	.29	−0.11, 0.03
SNOE	0.03	.54	−0.06, 0.12	−0.21	.00	−0.27, − 0.14	0.06	.05	0.00, 0.13

The significant background variables were controlled in the analyses. They were not presented because of page limits. Abbreviations: PPOE = anticipated physical benefits; RBD = Repeated blood donation; SEPOE = self-worth expectancy; PNOE = anticipated physical harms; SNOE = unfavorable social expectancies; SPOE = anticipated social or tangible gains.

### The moderation role of altruism

For brevity, only the primary model (the effect of overall outcome expectations) was discussed in detail in the main text; the 5 exploratory models can be found in the Supplementary Materials. Specifically, the moderated mediation model of anticipated physical benefits was significant (see Tables 2 and 3 and Supplementary Materials).

The results of the primary model showed a satisfactory model fit: *χ*^2^/df = 1.95, RMSEA = 0.033 (0.002, 0.058), CFI = 0.98, TLI = 0.92, SRMR = 0.045. Consistent with H3, results showed that the indirect effects of overall outcome expectations on RBD behavior through re-donation intention was moderated by altruism (Index of moderated mediation= − 0.04, *P < *.05). In addition, the total effect of overall outcome expectations on RBD behavior was significant among donors with lower levels of altruism (*B* = .12, *P < *.05) but non-significant among donors with middle and higher levels of altruism (*B* = .08, *P* = .15; *B* = 0.04, *P* = .48).

Specifically, overall outcome expectations were positively related to re-donation intention (*B* = 0.33), and altruism was positively related to re-donation intention (*B* = 0.16). A significant interaction effect of overall outcome expectations and altruism on re-donation intention was revealed (*B* = −0.09) (see Table 5). This suggests that the moderation effect of altruism between overall outcome expectations and re-donation intention was significant.

**Table 5:** Moderated mediation effect analyses for the role of altruism in the relationship between outcome expectations and repeated blood donation behavior through re-donation intention.

	Equation 1			Equation 2		
	(RBD intention)			(RBD behavior)		
	*B*	*SE*	*P value*	*B*	*SE*	*P value*
Outcome expectations × Altruism	−0.09	0.03	<.001			
Outcome expectations	0.33	0.03	<.001	−0.07	0.05	.21
Altruism	0.16	0.03	<.001	−0.06	0.05	.23
RBD Intention				0.39	0.05	<.001
Number of past donations	0.26	0.03	<.001	0.24	0.05	<.001
BMI	0.09	0.03	.01	0.08	0.05	.07
Married	0.01	0.04	.78	0.14	0.05	.01
Age	0.02	0.05	.65			
Student or unemployment	−0.13	0.05	.01			
IPFF				0.05	0.05	.29
2000 or less monthly income	0.04	0.05	.34			
4001 or more monthly income	−0.01	0.05	.81			
High school or below education	0.05	0.04	.21			
Residence	0.05	0.03	.12			
R^2^	0.279			0.297		
F	18.73			29.34		

IPFF = Individual practitioner or freelancer or farmer. RBD = Repeated blood donation. BMI, Age are continuous variables. The categorical demographic variables and RBD behavior were dummy coded.

**Figure 2.**
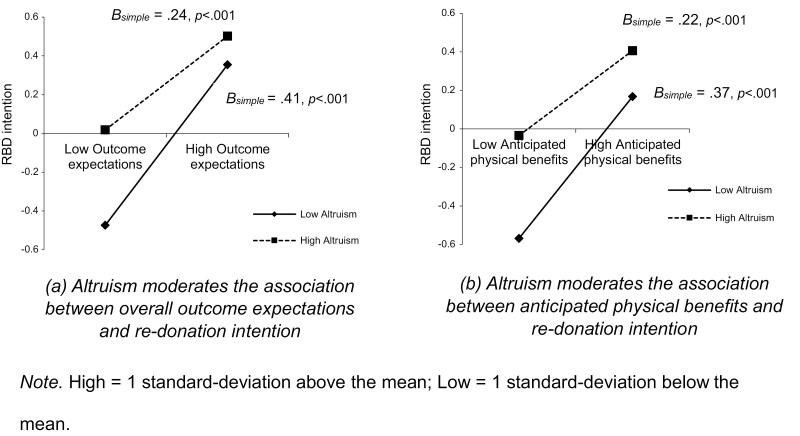
Altruism moderates the association between overall outcome expectations, anticipated physical benefits and re-donation intention.

The simple-slope tests denoted that the positive relationships between overall outcome expectations and re-donation intention as well as between anticipated physical benefits and re-donation intention were significantly stronger for blood donors with a lower level of altruism than for those with a higher level of altruism (see Figure 2). H3 was therefore supported.

## Discussion

The present study examined whether and how outcome expectations predict RBD behavior among Chinese blood donors. As hypothesized, the findings reveal that the positive influence of overall outcome expectations and anticipated physical benefits on RBD behavior was fully mediated by re-donation intention. Furthermore, altruism moderated the influence of overall outcome expectations and anticipated physical benefits on RBD behavior through re-donation intention. Specifically, the influence of overall outcome expectations and anticipated physical benefits on re-donation intention was stronger among donors with lower levels of altruism. The findings highlight the importance of enhancing overall outcome expectations, anticipated physical benefits of RBD, and altruism among donors.

### Anticipated physical benefits predicted more RBD behavior while anticipated physical harms predicted less RBD behavior

According to the prominent theories (social cognitive theory and theory of planned behavior) and literature, cognitive factors can have a direct or indirect effect on behavior through behavioral intention.^7,10,16,36^ In other words, the association between outcome expectations and RBD behavior can occur through a full mediator or a partial mediator. The present study found that the direct effect of overall outcome expectations on RBD behavior was non-significant after controlling for significant background variables, suggesting a full mediation. H1 was therefore not supported. It might be plausible that the influence of overall outcome expectations on RBD behavior occurred indirectly through other factors such as re-donation intention. Previous theoretical and empirical studies have suggested intention as an important mediator of the relationship between cognitive factors and behavior.^10,16,37^ Individuals’ positive outcome expectations must be translated into specific behavioral intentions in order to overcome practical barriers to action (eg, time constraints).^15^ The finding that re-donation intention fully mediated this relationship supports this claim. It might also be plausible that the relationship between overall outcome expectations and RBD behavior was only evident among certain groups of blood donors (eg, those with lower altruism). Previous literature has revealed that personal characteristics (eg, altruism, conscientiousness) are moderators that amplify or mitigate the impact of cognitive factors (eg, attitude, self-efficacy) on behavior intentions/actions.^20,21,38^

Among the different types of outcome expectations, we found that the anticipated physical benefits predicted more while anticipated physical harms predicted less RBD behavior. H1a and H1b were supported. This is consistent with theories (ie, SCT and health action process approach) and previous literature suggesting that positive outcome anticipations motivate behavior while negative ones deter behavior.^7,9,39^ The findings enrich the content/dimensions and understanding of significant positive and negative outcome expectations of RBD behavior. They additionally offer specific avenues for interventions or public health campaigns aimed at increasing blood donation (e., through improving anticipated physical benefits and reducing anticipated physical harms). To this end, it may be helpful to provide a role model, which has been evidenced to promote outcome expectations and prosocial behavior.^40^

It is interesting to note that the direct relationship between anticipated social or tangible gains, self-worth expectancies, and unfavorable social expectancies and RBD behavior were non-significant. This suggests that physical benefits and harms play a more unique and critical role in influencing RBD behavior than social or psychological factors (such as anticipated social gains and self-worth). According to protection motivation theory,^41^ individuals tend to perform threat appraisals and avoid engaging in behaviors that may risk their health. The perceived risks of blood donation (eg, infection, harm to health) can cause hesitation to re-donate.^13,42^ Also, blood donors may place more emphasis on physical consequences than social and psychological results.^43,44^ Therefore, compared with physical consequences, social or self-worth benefits or unfavorable expectancies might not be sufficient to motivate re-donation behavior.

### The mediation effect of re-donation intention

Overall outcome expectations and their 5 dimensions were associated with re-donation intention, which in turn predicted RBD behavior. H2 was supported. Specifically, RBD intention played a full mediation role between the 3 subscales (anticipated social or tangible gains, self-worth expectancies, and unfavorable social expectancies) and RBD behavior. Further, RBD intention played a partial mediation role between the 2 subscales (physical benefits and harms) and RBD behavior. The findings are consistent with theory of planned behavior and empirical literature suggesting that intention is a mediator in the association between social cognitive factors and RBD behavior.^10,16,17^ Outcome expectation involves the process in which donors evaluate the overall benefits and costs of RBD and decide to re-donate when the overall benefits outweigh the costs.^8,9,11^ It is therefore reasonable that outcome expectations of RBD can motivate re-donation intention. These findings suggest that RBD decision-making is a conscious, analytical process,^45^ with donors taking action only when their intention is sufficiently motivated. The findings also expand the theory of planned behavior by adding overall outcome expectations and the 5 subdimensions as proximal factors of intention and distal factors of RBD behavior.

### The moderating effect of altruism

The present study found that altruism moderated the association between overall outcome expectations and RBD behavior through re-donation intention, and between anticipated physical benefits and RBD behavior through re-donation intention. H3 was supported. Specifically, the positive relationships between overall outcome expectations and re-donation intention, and between anticipated physical benefits and re-donation intention were stronger among donors with lower (relative to higher) altruism. This may be because donors with higher altruism prioritize group/other's interests over their personal benefits; therefore, the influence of overall outcome expectations and anticipated physical benefits (that benefit oneself more) on re-donation intention/behavior (which benefits others more) is weaker in this group.

Notably, the impact of overall outcome expectations and anticipated physical benefits on RBD behavior was significant only among donors with lower altruism levels. This is a novel finding in revealing whether and when outcome expectations predicted RBD behavior among Chinese blood donors. This result suggests the importance of targeting for improving outcome expectations and anticipated physical benefits in intervention programs for donors with lower levels of altruism. Interventions aimed at promoting altruism would also be helpful; these could include incorporating relatedness primes—that is, increasing one's feelings of connectedness to others within donation campaign materials; simple moral nudges, that is, asking individuals “what's the morally right thing to do?” before they make actual choices; watching a video evoking moral elevation, enacting prosocial behaviors, and reflecting on how those behaviors relate to one's values, among others.^46-48^

### Limitations and future study directions

The current study has some limitations. First, the 6-month follow-up period may be insufficient to fully capture RBD behavior. Longitudinal studies with longer observational times, such as 12 months or 24 months, may be considered in future research. Second, some data (ie, altruism, outcome expectations, and intentions) are cross-sectional, limiting the ability to examine causality between outcome expectations and re-donation intention. Third, although the follow-up measurement used an objective method to capture the RBD behavior, self-reported data at baseline may introduce response bias. Future studies may include objective measures or other-rating measures (eg, scheduling a donation appointment for the re-donation intention variable; multiple sources of altruism data such as from friends or family members) at baseline. Fourth, although the sample characteristics were generally representative of Chinese blood donors,^49-51^ the low response rate for the online survey may limit the generalizability of the findings.

### Implications and conclusion

Given current blood shortages and the increasing demand for blood,^52,53^ it is crucial to examine factors influencing RBD and outline strategies to improve blood donation rates. This is the first study to examine the joint effects of outcome expectations and altruism on re-donation intention/behavior, revealing the mechanisms underlying how and when overall outcome expectations promote RBD.

The results of this study enrich our understanding of RBD as both a health and prosocial behavior and provide valuable insights for designing effective interventions. First, the stronger association between outcome expectations and re-donation intention among donors with lower altruism suggests that more attention should be paid to enhancing positive outcome expectations and reducing negative ones for donors in this group. Social marketing messages highlighting the social, physical, and self-evaluative outcomes and benefits of RBD may be effective. Of the various dimensions of outcome expectations, anticipated physical benefits should be highly emphasized in social marketing media. Second, fostering altruism may be particularly effective in promoting re-donation intention among donors with lower levels of overall outcome expectations and anticipated physical benefits. Empirical studies have reported that highlighting relatedness, moral nudges, combined interventions involving moral elevation, prosocial behavior, and values identification may enhance altruism, donation motives, and donation behavior among blood donors.^46-48^

## Supplementary Material

kaaf036_suppl_Supplementary_Tables_1-3

## Data Availability

De-identified data from this study are not available in public archive. De-identified data from this study will be made available (as allowable according to institutional IRB standards) by emailing the corresponding author. Analytic code availability: Analytic code used to conduct the analyses presented in this study are not available in a public archive. They may be available by emailing the corresponding author. Materials availability: Materials used to conduct the study are not publically available.
