# Supplementary material for: Outcome expectations and repeated blood donation behavior: a moderated mediation model in a prospective observational study
Source: Ann Behav Med. 2025 May 21;59(1):kaaf036. doi: 10.1093/abm/kaaf036 (PMC12169329; doi:10.1093/abm/kaaf036)
Supplement: kaaf036_suppl_Supplementary_Tables_1-3 [file kaaf036_suppl_supplementary_tables_1-3.docx]

Supplementary Materials

**Table 1**

*Donation information of blood donors since 2018 and the percentage of participants*

| Type | 2018 | 2019 | July 2020 to Apr 2021^a^ | Number of donors randomly selected | % (N) | Note |
| --- | --- | --- | --- | --- | --- | --- |
| 1 | first donation | not return | not return | 3200 | 9% (75) | Donors who made their first donation in 2018 and not return afterwards |
| 2 | first donation | re-donate | not return | 3200 | 16% (140) | Donors who made their first donation in 2018, returned in 2019 but not return afterwards |
| 3 | first donation | not return | re-donate | 2967^b^ | 22% (184) | Donors who made their first donation in 2018, not return in 2019 but return during July 2020 to Apr 2021 |
| 4 | / | first donation | not return | 3200 | 13% (106) | Donors who made their first donation in 2019 but not return afterwards |
| 5 | / | first donation | re-donate | 3200 | 27% (228) | Donors who made their first donation in 2019 and return afterwards |
| 6 | / | / | first donation | 3200 | 14% (117) | Donors who made their first donation during July 2020 to Apr 2021 |
| Total |  |  |  | 18967 | 100% (850) |  |

*Note.* a. Donation behavior during February-June 2020 was not considered as China was heavily affected by the covid-19 epidemic during this period; b. Only 2967 donors could be located for this type of donors in the database.

**Table 2**

*Conditional direct and indirect effect of outcome expectations on repeated blood donation behavior via re-donation intention*

|  |  | Z score of | Effect | SE | P value |
| --- | --- | --- | --- | --- | --- |
|  |  | Altruism |  |  |  |
| Total OE |  |  |  |  |  |
| Conditional indirect effect | | -1 | 0.18 | 0.03 | 0.00 |
|  |  | 0 | 0.14 | 0.02 | 0.00 |
|  |  | 1 | 0.11 | 0.02 | 0.00 |
| Index of moderated mediation: | | | -0.04 | 0.01 | 0.01 |
| Total effect | | -1 | 0.11 | 0.06 | 0.07 |
|  |  | 0 | 0.07 | 0.06 | 0.21 |
|  |  | 1 | 0.03 | 0.06 | 0.56 |
| PPOE |  |  |  |  |  |
| Conditional indirect effect | | -1 | 0.16 | 0.03 | 0.00 |
|  |  | 0 | 0.13 | 0.02 | 0.00 |
|  |  | 1 | 0.09 | 0.02 | 0.00 |
| Index of moderated mediation: | | | -0.03 | 0.01 | 0.02 |
| Total effect | | -1 | 0.14 | 0.05 | 0.01 |
|  |  | 0 | 0.11 | 0.05 | 0.03 |
|  |  | 1 | 0.08 | 0.05 | 0.14 |
| SPOE |  |  |  |  |  |
| Conditional indirect effect | | -1 | 0.10 | 0.02 | 0.00 |
|  |  | 0 | 0.09 | 0.02 | 0.00 |
|  |  | 1 | 0.08 | 0.02 | 0.00 |
| Index of moderated mediation: | | | -0.01 | 0.01 | 0.41 |
| Total effect | | -1 | 0.07 | 0.05 | 0.18 |
|  |  | 0 | 0.06 | 0.05 | 0.23 |
|  |  | 1 | 0.05 | 0.05 | 0.33 |
| SEPOE |  |  |  |  |  |
| Conditional indirect effect | | -1 | 0.12 | 0.02 | 0.00 |
|  |  | 0 | 0.10 | 0.02 | 0.00 |
|  |  | 1 | 0.08 | 0.02 | 0.00 |
| Index of moderated mediation: | | | -0.02 | 0.01 | 0.19 |
| Total effect | | -1 | 0.11 | 0.06 | 0.06 |
|  |  | 0 | 0.09 | 0.06 | 0.10 |
|  |  | 1 | 0.08 | 0.06 | 0.19 |
| PNOE |  |  |  |  |  |
| Conditional indirect effect | | -1 | -0.10 | 0.02 | 0.00 |
|  |  | 0 | -0.07 | 0.02 | 0.00 |
|  |  | 1 | -0.05 | 0.02 | 0.03 |
| Index of moderated mediation: | | | -0.01 | 0.01 | 0.08 |
| Total effect | | -1 | -0.12 | 0.06 | 0.03 |
|  |  | 0 | -0.10 | 0.05 | 0.06 |
|  |  | 1 | -0.08 | 0.05 | 0.16 |
| SNOE |  |  |  |  |  |
| Conditional indirect effect | | -1 | -0.09 | 0.02 | 0.00 |
|  |  | 0 | -0.08 | 0.02 | 0.00 |
|  |  | 1 | -0.06 | 0.02 | 0.00 |
| Index of moderated mediation: | | | 0.01 | 0.01 | 0.32 |
| Total effect | | -1 | 0.03 | 0.06 | 0.58 |
|  |  | 0 | 0.04 | 0.05 | 0.43 |
|  |  | 1 | 0.06 | 0.05 | 0.31 |

*Note.* PPOE= anticipated physical benefits; SPOE = anticipated social or tangible gains; SEPOE =self-worth expectancy; PNOE =anticipated physical harms; SNOE= unfavorable social expectancies.

**Table 3**

*Moderated mediation effect analyses for the role of altruism in the relationship between five dimensions of outcome expectations and RBD behavior through RBD intention*

|  | Equation 1 | | | Equation 2 | | |
| --- | --- | --- | --- | --- | --- | --- |
|  | (RBD intention) | | | (RBD behavior) | | |
|  | *B* | *SE* | *p value* | *B* | *SE* | *p value* |
| PPOE × Altruism | -0.07 | 0.03 | 0.01 |  |  |  |
| PPOE | 0.30 | 0.03 | 0.00 | -0.01 | 0.05 | 0.77 |
| Altruism | 0.19 | 0.03 | 0.00 | -0.07 | 0.05 | 0.14 |
|  |  |  |  |  |  |  |
| SPOE × Altruism | -0.03 | 0.03 | 0.40 |  |  |  |
| SPOE | 0.22 | 0.03 | 0.00 | -0.03 | 0.05 | 0.53 |
| Altruism | 0.21 | 0.03 | 0.00 | -0.06 | 0.05 | 0.18 |
|  |  |  |  |  |  |  |
| SEPOE × Altruism | -0.04 | 0.03 | 0.19 |  |  |  |
| SEPOE | 0.24 | 0.03 | 0.00 | -0.01 | 0.05 | 0.87 |
| Altruism | 0.18 | 0.03 | 0.00 | -0.06 | 0.05 | 0.18 |
|  |  |  |  |  |  |  |
| PNOE × Altruism | 0.06 | 0.03 | 0.07 |  |  |  |
| PNOE | -0.17 | 0.03 | 0.00 | -0.02 | 0.05 | 0.61 |
| Altruism | 0.22 | 0.03 | 0.00 | -0.08 | 0.05 | 0.08 |
|  |  |  |  |  |  |  |
| SNOE × Altruism | 0.03 | 0.03 | 0.31 |  |  |  |
| SNOE | -0.17 | 0.03 | 0.00 | 0.11 | 0.05 | 0.03 |
| Altruism | 0.22 | 0.03 | 0.00 | -0.05 | 0.05 | 0.30 |

*Note.* The significant background variables were controlled in the analyses. They were not presented due to page limits. RBD= Repeated blood donation. PPOE= anticipated physical benefits; SPOE = anticipated social or tangible gains; SEPOE =self-worth expectancy; PNOE =anticipated physical harms; SNOE= unfavorable social expectancies.
